# Polyphenol Health Effects on Cardiovascular and Neurodegenerative Disorders: A Review and Meta-Analysis

**DOI:** 10.3390/ijms20020351

**Published:** 2019-01-16

**Authors:** Francesco Potì, Daniele Santi, Giorgia Spaggiari, Francesca Zimetti, Ilaria Zanotti

**Affiliations:** 1 Dipartimento di Medicina e Chirurgia, Unità di Neuroscienze, Università di Parma, via Volturno 39/F, 43125 Parma, Italy; francesco.poti@unipr.it; 2 Dipartimento di Scienze Biomediche, Metaboliche e Neuroscienze, Unità di Endocrinologia, Università degli Studi di Modena e Reggio Emilia, via del Pozzo 71, 41124 Modena, Italy; daniele.santi@unimore.it; 3 Dipartimento di Medicine Specialistiche-Unità di Endocrinologia, Azienda Ospedaliero-Universitaria di Modena, Ospedale Civile di Baggiovara, via Giardini 1355, 41126 Modena, Italy; giorgia.spaggiari87@gmail.com; 4 Dipartimento di Scienze degli Alimenti e del Farmaco, Università di Parma, Parco Area delle Scienze 27/A, 43124 Parma, Italy; francesca.zimetti@unipr.it

**Keywords:** polyphenols, prevention, cardiovascular disease, neurodegenerative disease, cognitive decline, meta-analysis, blood pressure, flow mediated dilation, low density lipoproteins, memory

## Abstract

Several studies have demonstrated that polyphenol-enriched diets may have beneficial effects against the development of degenerative diseases, including atherosclerosis and disorders affecting the central nervous system. This activity has been associated not only with antioxidant and anti-inflammatory properties, but also with additional mechanisms, such as the modulation of lipid metabolism and gut microbiota function. However, long-term studies on humans provided controversial results, making the prediction of polyphenol impact on health uncertain. The aim of this review is to provide an overview and critical analysis of the literature related to the effects of the principal dietary polyphenols on cardiovascular and neurodegenerative disorders. We critically considered and meta-analyzed randomized controlled clinical trials involving subjects taking polyphenol-based supplements. Although some polyphenols might improve specific markers of cardiovascular risk and cognitive status, many inconsistent data are present in literature. Therefore, definitive recommendations for the use of these compounds in the prevention of cardiovascular disease and cognitive decline are currently not applicable. Once pivotal aspects for the definition of polyphenol bioactivity, such as the characterization of pharmacokinetics and safety, are addressed, it will be possible to have a clear picture of the realistic potential of polyphenols for disease prevention.

## 1. Introduction

Interest in the health effects of polyphenols has exponentially increased in recent years. Since the 80s, more than 80,000 studies have been published in PubMed, reporting multiple effects on a broad range of diseases [1]. However, most of this evidence was derived from in vitro or animal studies [2], while human trials that evaluate long-term effects of polyphenols are particularly scant. Moreover, no studies with hard clinical end points have been conducted so far [3]. The main limitations of current published studies include inaccuracy in the selection of tested concentrations. In fact, much higher amounts of compounds than those found in dietary sources are often analyzed. In addition, the lack of information on pharmacokinetics (absorption and metabolism) may cause the choice of compounds that are different from those actually bioavailable. In addition, the studies aimed at identifying cellular targets involved in the beneficial effects of dietary polyphenols challenge the translatability of these data. In fact, polyphenols have low bioavailability, related to the appearance in food as glycosylated compounds with a very limited intestinal absorption. However, once in the colon, enzymatic cleavage by the microbiota releases aglycon, which could undergo absorption or additional metabolism [4]. Thus, while most studies demonstrating polyphenol mechanisms have been carried out in cultured cells, effective concentrations and effective bioactive compounds are often not tested, challenging the physiological relevance of these observations. As a consequence, a lot of data resulting from published reports have little relevance in real life, with the exception of those describing beneficial effects of polyphenols from cocoa and olive oil [5].

The health effects of polyphenols for the prevention of several disturbances ranging from cancer to metabolic syndrome, diabetes, non-alcoholic liver disease and periodontal disease have been reported. In the present review, we critically analyzed the literature concerning polyphenol activity on human cardiovascular and neurodegenerative diseases. We focused on cardiovascular and neurodegenerative diseases, whose high incidence in the world represents an increasing cause of mortality, morbidity, and disability. We selected studies on humans taking polyphenol-enriched dietary supplements for a preventive purpose, avoiding those based on dietary approach. Although this methodological strategy diverges from real life, the choice to focus on determined groups of polyphenols allows us to more accurately evaluate their biological effects, without the confounding presence of other bioactive food components and food matrices.

## 2. Polyphenol Effects on Cardiovascular Disease (CVD)

### 2.1. Impact of Polyphenols on Cardiovascular Disease in Humans

Globally, CVD is the major cause of morbidity and mortality, accounting for 32% of all causes and for 44% of all non-communicable disease deaths. Of these deaths, 85% are due to ischemic heart disease and stroke [6]. In the last 18 years, the risk of dying from cardiovascular disease or other non-communicable diseases showed a slight, but constant decrease. This trend could be caused by the implementation of several preventive measures, including actions to reduce key risk factors such as unhealthy diet and alcohol consumption, physical inactivity, tobacco use, and constant exposure to air pollution [7]. The pharmacological treatment of CVD has also greatly improved life expectancy and quality, in either primary or secondary prevention, but side-effects and limited adherence to treatments have often dampened drug effectiveness. In addition to pharmacological therapy, dietary supplements, functional foods and nutraceutical products are being increasingly used for cardiovascular health, despite the lack of high-quality human trials evaluating the efficacy of such additional interventions [8,9]. Based on the recent recommendations for high-quality polyphenol research [10] and with the aim to provide a critical review of the recent evidence in the cardiovascular field, we herein focused exclusively on intervention studies, conducted in primary prevention, and in which polyphenols were taken as nutraceuticals or other titrated formulations. Two excellent reviews extensively treated the impact of common polyphenol-based nutraceuticals and dietary products on cardiovascular health [2,3]; readers are invited to refer to them for a comprehensive reading. In the present work, only trials evaluating the effects of the administration of polyphenol-enriched extracts or isolated compounds were considered, while those contemplating polyphenol-rich food consumption, such as tea, coffee, chocolate or fruit, in any formulations (e.g., whole fresh or dried product, beverages, etc.), or multiple compound-enriched preparations, were excluded. Particular attention was paid to relatively long (longer than two weeks) studies and those with well-defined cardiovascular endpoints. Systematic and quantitative analysis of data from the selected studies are described in the meta-analysis section, while the following narrative part discusses multiple endpoints from recent (last five years) trials.

Amongst all the investigated CVD hallmarks, hypertension, dyslipidemia, endothelial dysfunction and systemic inflammation were the most reported in selected literature. Specific markers have been used for evaluating cardiovascular health. Increased total or low density lipoprotein cholesterol (LDL-C), or diminished high density lipoprotein cholesterol (HDL-C) levels, are consolidated markers of increased cardiovascular risk and directly contribute to the atherosclerotic plaque physiopathology [11]. In addition, other proteins like apolipoprotein A–I or paraoxonase-1, which are constitutive components of HDL particles, mainly displaying atheroprotective effects, have been inversely correlated with cardiovascular risk. Specific markers of endothelial activation and dysfunction, causal drivers of vascular damage, may be measured both in plasma and through minimally invasive ultrasound techniques. For example, endothelin-1, soluble endothelial-derived adhesion molecules (sICAM-1, sVCAM-1), asymmetric dimethylarginine, exerting pro-inflammatory actions on vascular endothelium, as well as high-sensitive C-reactive protein (hs-CRP), tumor necrosis factor alpha (TNF-α) and interleukin-1 beta, systemic inflammation markers, are commonly used [12,13]. Moreover, the brachial artery flow mediated dilation (FMD) has found wide application. It is defined as the percent change in brachial artery diameter, following to reactive hyperaemia, typically induced by a five-minutes circulatory arrest through a supra-systolic cuff occlusion, causing NO release and vasodilation [14].

However, high heterogeneity in experimental design, study population, compounds and endpoints evaluated, as well as the paucity of data about the biological activity of the different metabolites hampered the possibility of clear comparisons or univocal conclusions.

A small randomized, double-blind placebo-controlled clinical trial evaluated the effects of 200 mg/day of red grape seed extract-derived oligomeric proanthocyanidin complexes for eight weeks, on 70 mild to moderate hyperlipidemic subjects. Argani H. and colleagues [15] observed a significant reduction of plasma total cholesterol (−14.8 mg/dL ± 19.7 vs. baseline, *p* = 0.001), triglycerides (−19.4 mg/dL ± 42.4 vs. baseline, *p* = 0.001) and LDL-C (−13.1 mg/dL ± 20.6 vs. baseline, *p* = 0.002). In the same study, anti-atherogenic components of plasma, such as apolipoprotein A–I (9.3 mg/dL ± 11.7 vs. baseline, *p* = 0.001), paraoxonase-1 (4.5 IU/L ± 7.7 vs. baseline, *p* = 0.03) and HDL-C (2.1 mg/dL ± 3.7 vs. baseline, not significant) increased as a consequence of treatment.

In another study, 70 subjects were randomized to receive 162 mg/day of quercetin from onion peel extract or placebo in a double-blinded, placebo-controlled cross-over trial with six-week treatment periods separated by a six-week washout period [16]. This study is of particular interest because subjects were also controlled for plasma concentrations of quercetin, its monomethylated derivatives tamarixetin (4′-*O*-methyl quercetin), isorhamnetin (3′-*O*-methyl quercetin) and the dehydroxylated quercetin metabolite kaempferol. In the subgroup of the hypertensive subjects, quercetin significantly decreased systolic blood pressure (SBP) by −3.6 mmHg (*p* = 0.022) when compared with placebo (mean treatment difference, −3.9 mmHg; *p* = 0.049). Notwithstanding, vasoactive biomarkers including endothelin-1, sICAM-1, sVCAM-1, asymmetric dimethylarginine, angiotensin-converting enzyme activity, vascular/endothelial function (evaluated by peripheral arterial tonometry, a technology to assess the reactive hyperaemia index), parameters of oxidation, were not affected by quercetin in the total group and in the subgroup of hypertensives. In the same cohort [17], authors did not find any significant changes in serum concentrations of leptin and adiponectin, homeostasis model assessment-adiponectin (HOMA-AD), glucose, insulin, homeostasis model assessment of insulin resistance (HOMA-IR), blood biomarkers of liver and renal function, hematology, serum electrolytes, hs-CRP and plasma TNF-α. On the contrary, a randomized double-blind, placebo-controlled study involving 72 healthy, overweight, and obese participants, randomly assigned to receive 100 mg/day of quercetin for 12 weeks did not find any significant difference in blood pressure between treatment and placebo arm, nor versus baseline [18]. However, treatment was effective in ameliorating endothelial function evaluated by percent FMD (from 12.5 ± 5.2% to 15.2 ± 6.1%; *p* = 0.002), and circulating endothelial progenitor cells counts by flow cytometry (44.2 ± 25.6% vs. 52.3 ± 18.6%; *p* = 0.005), compared with the baseline values.

A randomized, double-blind, placebo-controlled crossover trial evaluated the effects of pure (−)-epicatechin (100 mg/day) and quercetin-3-glucoside (160 mg/day) on biomarkers of endothelial dysfunction and inflammation [19] or vascular function and cardiometabolic health [20], in a cohort of 37 apparently healthy pre-hypertensive (SBP = 125=−160 mmHg) men and women aged 40–80 years. The first analysis showed reduced plasma levels of markers of vascular inflammation, such as soluble endothelial-selectin or interleukin-1 beta. However, the differences were not significant in most cases.

In the second analysis, epicatechin supplementation improved fasting plasma insulin and HOMA-IR, without affecting blood pressure, arterial stiffness, nitric oxide (NO), endothelin-1, or blood lipid profile. Quercetin-3-glucoside supplementation had no effect on FMD, insulin resistance, or other CVD risk factors. Although the compliance and polyphenol absorption were monitored after four weeks of treatment by measuring plasma and urine epicatechin and quercetin concentrations, no further analysis of metabolites was carried out. However, studying pure flavonoids instead of the original complex matrixes may raise some important issues. In fact, if on the one hand, this approach reduces the burden of potential confounding factors, on the other, it may exclude potential favorable interactions with other flavonoids and compounds naturally present in original sources, like cocoa or tea.

### 2.2. Mechanisms of Cardioprotection

The role of oxidative stress as a promoter of endothelial dysfunction, that in turn is a driver of early atherosclerosis and consequent cardiovascular related disorders, is no longer in doubt and provides a support for the anti-inflammatory and anti-oxidant strategy in the field of cardioprotection [21]. The widely described antioxidant properties of polyphenols relies on the presence of hydroxyl groups that can be readily oxidized to produce the corresponding O-quinones [22]. This conversion results in an effective scavenger activity towards reactive oxygen species, which occurs through the entrapment of free radicals into stabilized chemical complexes, thus preventing further reactions [23]. This so-called “biochemical scavenger theory” is currently the most validated one to explain the beneficial effects towards a broad range of non-communicable diseases, including CVD. However, it has to be noted that, although the antioxidant capacity of polyphenols has been largely tested, the results of in vitro tests not always translate in an increased antioxidant status in humans. This lack of consistency may be due to high variability of the single in vitro assays, as well as to individual-related factors [24].

Beyond the inhibition of oxidative stress, polyphenols also display indirect antioxidant effects, occurring through the activation of the transcription nuclear factor erythroid 2-related factor 2 (Nrf2). This event induces endogenous antioxidant systems and is likely to be responsible also for polyphenol-mediated maintenance of the correct redox balance of cells, achieved through the equilibrium of phase I and II enzyme activity [25]. The anti-inflammatory properties of polyphenols are strictly connected with the modulation of oxidative stress and of the balance of redox cellular homeostasis [26]. Multiple mechanisms, most of which are mediated by the inhibition of the nuclear factor kappa-light-chain-enhancer of activated B cells (NF-kB), account for polyphenol’s anti-inflammatory activity. These compounds are able to decrease the cellular production of pro-inflammatory mediators [27,28,29] and to inhibit the expression of adhesion molecules [30], thus impairing the chemotaxis of monocytes within the inflamed tissues. Additional mechanisms accounting for the cardioprotective action of polyphenols target lipid metabolism, whose impairment represents a causative factor of atherosclerosis development [31]. An exhaustive appraisal of polyphenol-enriched food capacity to beneficially modulate lipid and lipoprotein metabolism has been recently reviewed by our group [32]. The widely reported decrease of total and LDL-C following the intake of polyphenols is possibly related to mechanisms occurring at hepatic and intestinal level. In the former, the reduction of cholesterol synthesis, the increase of LDL receptor expression and activity [33] and the increase of the cholesterol transporters ATP-Binding Cassette G5/ATP-Binding Cassette G8 expression [34] have been described in in vivo models. In the latter, polyphenol (i.e., epigallocatechin gallate) capacity to displace cholesterol from intestinal micelles have been associated with increased cholesterol fecal elimination in vivo [35]. The effect on triglyceride plasma level is possibly related to the reduction of apolipoprotein B48 and apolipoprotein B100 production in the liver and intestine, as demonstrated in obese subjects [36], or to the interference with lipoprotein lipase expression, as evidenced in pigs [37]. The mechanisms accounting for the increase of HDL-C are limited to in vitro evidence. Among them, the increase in apolipoprotein A–I synthesis has been reported in cultured hepatic or intestinal cells exposed to cocoa polyphenols [38].

Polyphenols could also be able to positively affect endothelial function, whose impairment is an established key factor for the development of atherosclerosis. This activity has been demonstrated as an amelioration of FMD in humans, although the effective dose was significantly higher than the typical dietary intake [39]. The mechanism accounting for improved FMD probably relies on the increase of NO synthase activity, as suggested by in vitro [40] and human studies [41]. This NO-mediated vasodilation, together with the influence on the renin-angiotensin system [42], is responsible for the reduction of blood pressure, an additional cardioprotective activity of polyphenols. Interestingly, this effect is also evident upon consumption of low, habitual amount of polyphenol-rich food [43].

Finally, polyphenols cardiovascular benefit may be ascribed to the peculiar pharmacokinetic properties mentioned above. While these compounds mainly reach the distal tract of gastrointestinal system unchanged, once modified by the gut microbiota, they may exert a prebiotic-like activity [44], causing the selective growth of beneficial bacteria [45,46,47], together with the inhibition of harmful strains [46,47,48]. This effect has been demonstrated in intervention studies, as well as in ex vivo models of fecal fermentation, possibly accounting for the amelioration of markers of CVD.

### 2.3. Meta-Analysis

#### 2.3.1. Methods

The literature search was performed evaluating both MEDLINE and Embase databases, considering all manuscript published in English language until November 10, 2018. The following search strategy was performed: ((polyphenol) OR flavonoids) OR polyphenolic compounds) OR isoflavone) OR flavanol) OR phytoestrogen) OR resveratrol) AND atherosclerosis) OR cardiovascular) OR cardiovascular disorder) OR cardiovascular health. The literature search was repeated adding the words “administration” and “therapy” using Mesh terms. Inclusion criteria were: (i) double-blind, randomized, controlled study design; (ii) adult patients (older than 18 years); (iii) chronic polyphenol administration; (iv) administration of polyphenols as food supplement or isolated compounds. Thus, both sexes were considered eligible, as well as all polyphenol compounds and route of administration. Moreover, only treatment periods over 2 weeks were included. During data extraction, the following endpoints were analyzed: FMD, LDL-C, HDL-C, hs-CRP and blood pressure.

The meta-analysis of the data extracted from the included studies was conducted using the Review Manager 5.2 software (Version 5.2.4 Copenhagen: The Nordic Cochrane Centre, The Cochrane Collaboration, 2012). Data were combined using random or fixed effect models, according to data heterogeneity. Heterogeneity between the results of different studies was examined by inspecting the scatter in the data points and the overlap in their confidence intervals and by performing chi-square tests and I^2^ statistics. When I^2^ was higher than 60%, a random effect model was used since it provides a more conservative estimate of the overall effect, especially relevant when studies were of different design and duration. Weighted mean differences and 95% confidence intervals were estimated for each endpoint considered. Values of *p* < 0.05 were considered statistically significant.

#### 2.3.2. Results

**Figure 1:**
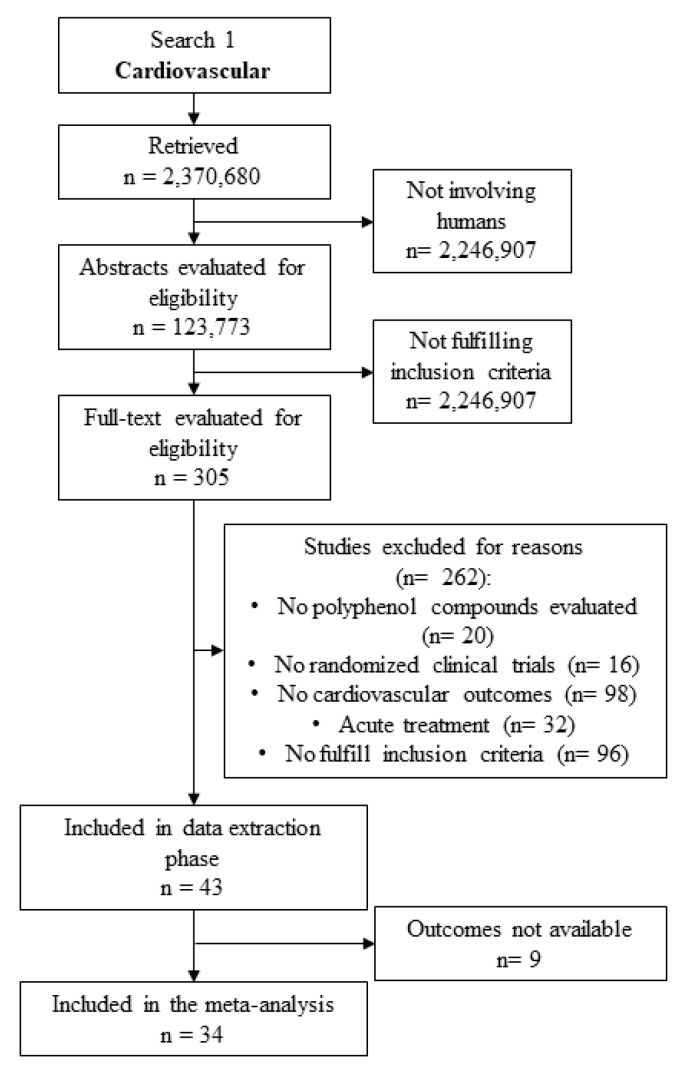
Literature search flow chart for meta-analysis on cardiovascular outcomes.

The flow chart of literature search is depicted in Figure 1.

We initially detected a total of 2,370,680 studies. Once only studies involving humans in a clinical trial were considered, an amount of 123,773 studies was selected. Among these, 43 studies were evaluated to extract cardiovascular data. Finally, 9 studies were excluded (Table S1), while 34 studies were included in the meta-analysis (Table 1).

**Figure 2:**
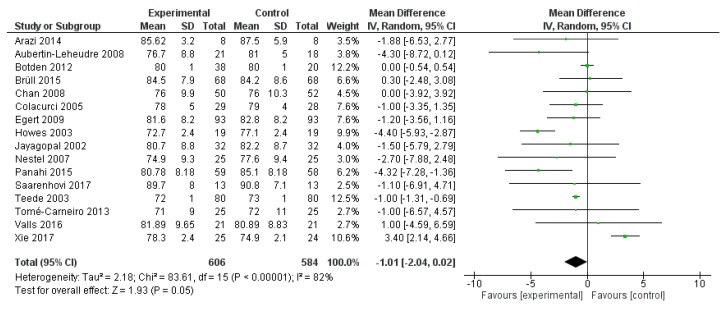
Pooled analysis of association between polyphenol intake and systolic blood pressure (SBP). The green square represents the mean difference detected in each study. The black diamond shows the final overall mean difference.

SBP was reported in 16 studies, evaluating a total of 1190 subjects (606 treated with polyphenols and 584 with placebo). The overall analysis showed a significant systolic pressure reduction after polyphenol administration (mean difference −1.01, 95%CI: −2.04; 0.02, *p* = 0.005) (Figure 2).

**Figure 3:**
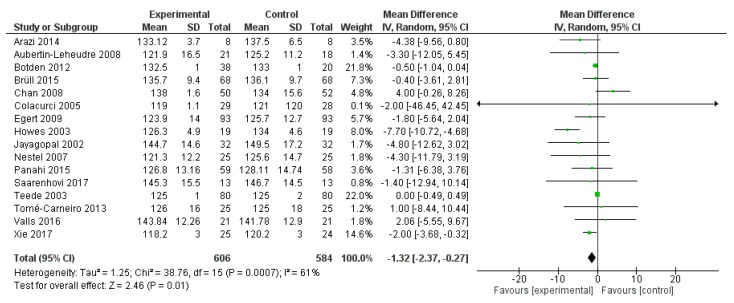
Pooled analysis of association between polyphenol intake and diastolic blood pressure.

In the same studies, diastolic blood pressure was significantly reduced by polyphenol administration (mean difference −1.32 mmHg, 95% CI: −2.37, −0.27 mmHg; *p* = 0.001) (Figure 3).

**Figure 4:**
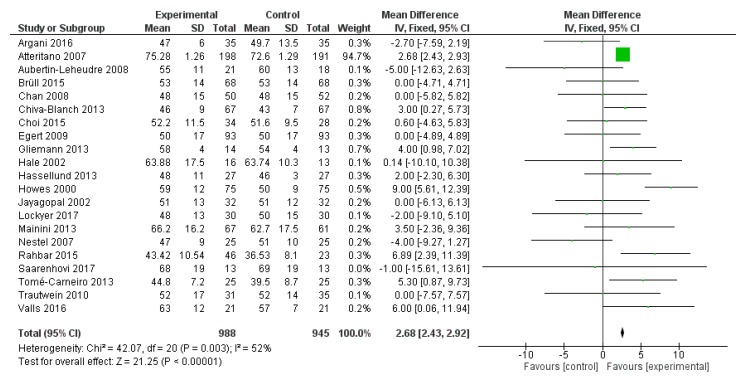
Pooled analysis of association between polyphenol intake and high-density lipoprotein cholesterol (HDL-C).

HDL-C was reported in 21 studies, for a total of 1933 patients (988 treated with polyphenols and 945 with placebo). The polyphenol administration significantly increased HDL-C serum levels (mean difference 2.68 mg/dL, 95% CI: 2.43, 2.92 mg/dL, *p* < 0.001) (Figure 4).

**Figure 5:**
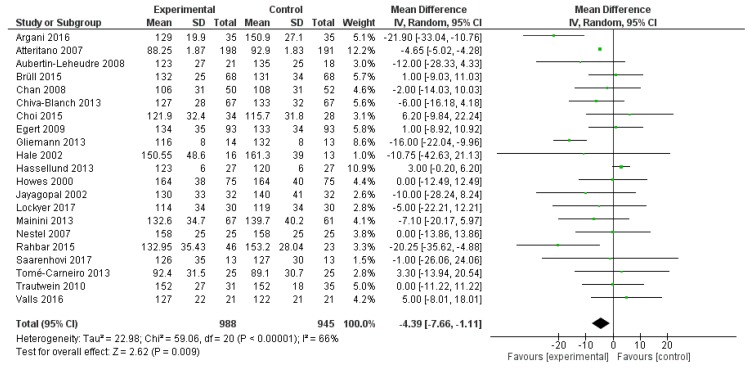
Pooled analysis of association between polyphenol intake and low-density lipoprotein cholesterol (LDL-C).

Similarly, LDL-C serum levels were significantly reduced after polyphenol administration (mean difference −4.39 mg/dL, 95%CI: −7.66, −1.11 mg/dL, *p* = 0.009) (Figure 5).

**Figure 6:**
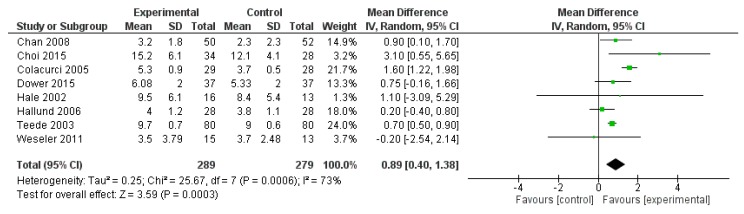
Pooled analysis of association between polyphenol intake and flow mediated dilation (FMD).

FMD was reported in eight studies, for a total of 568 patients (289 treated with polyphenols and 279 with placebo). The polyphenol administration significantly increased FMD (mean difference 0.89%, 95% CI: 0.40, 1.38%, *p* < 0.001) (Figure 6).

**Figure 7:**
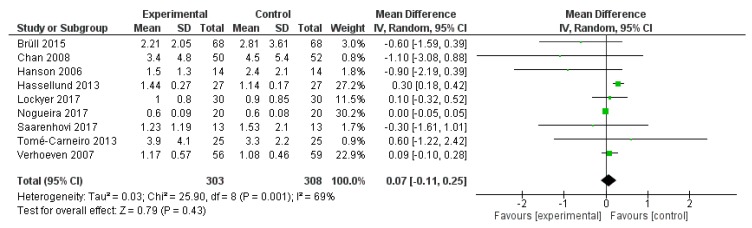
Pooled analysis of association between polyphenol intake and high-sensitive C-reactive protein (hs-CRP).

Hs-CRP was reported in nine studies, for a total of 611 patients (303 treated with polyphenol and 308 with placebo). The polyphenol administration did not change hs-CRP plasma levels (*p* < 0.430) (Figure 7).

#### 2.3.3. Discussion

In an attempt to provide a quantitative assessment of the possible effects of polyphenols on cardiovascular health, we performed a meta-analysis of the main parameters evaluated in the selected studies. All the tested dosages tended to be higher than those reachable with a common diet, as our analysis specifically focused on polyphenol supplementation. This is particularly true for flavanols like quercetin (>100 mg/day) [16,17,18,19] and isoflavones (80 to 132 mg/day, Mainini 2013 and Jayagopal 2002), while in some cases, the doses of oligomeric proanthocyanidin complexes (200 mg/day [15]) ranged around those achievable by diet [49].

According to the inclusion and exclusion criteria, a satisfactory number of studies contributed to the analysis (17 for blood pressure, 21 for blood lipids and 9 for endothelial function and systemic inflammation) and the number of subjects evaluated ranges from 500 to 2000, for each parameter considered. However, high heterogeneity has been found, even because of the differences in the treatment, in terms of formulation, dose, source and identity of the evaluated polyphenol. These discrepancies hampered the possibility to carry out subgroup analysis. Notwithstanding, the overall analysis revealed a significant effect of polyphenols in positively modulating the cardiovascular parameters considered. Treatments lowered systolic and diastolic blood pressure, as well as plasma levels of LDL-C, while increasing HDL-C levels and percent FMD. Interestingly, the effect on blood pressure seems to be stronger in the diastolic than systolic one. Finally, no significant effects were detected for hs-CRP by our meta-analysis. Although these effects are significant from a statistical point of view, the detected differences are undoubtedly of modest size and their actual clinical benefit remains to be established. For example, the absolute reduction in LDL-C (mean difference: −4.39 mg/dL) may be of interest, since it has been shown that a linear relationship exists between LDL-C lowering and the rate of major vascular events [50]. The effect on HDL-C is far more complex to evaluate. In fact, the effect is due for about 95% to a single study [51] out of 21 total studies considered. Furthermore, according to the most recent evidences coming from both Mendelian randomization studies and clinical trials [52,53,54], increasing HDL-C in subjects with normal baseline values is not an effective approach for cardiovascular risk reduction. The value of these results can be further corroborated by a recent analysis, aimed at defining the appropriateness of outcome variables for cardiovascular health claims compliant with European Community Regulation 1924/2006 [55]. Accordingly, the study provided evidence of risk factors to be used for the substantiation of health claims in the context of CVD, such as LDL-C and SBP, while other factors, like HDL-C or triglycerides, were not recommended.

## 3. Polyphenol Effects on Neurocognitive Functions

### 3.1. Impact of Polyphenols on Cognitive Functions in Humans

The significant advances in medicine have brought about a marked increase in lifespan over the last years. Despite the obvious positive consequences, population aging led to a significant raise in the onset of age-associated diseases, including neurodegenerative diseases [56]. Neurodegeneration is a common condition shared by several disorders, such as amyotrophic lateral sclerosis, Huntington’s, Alzheimer’s disease (AD), frontotemporal dementia, and Parkinson’s disease [57]. Their etiology is multifactorial and still far from being fully understood. Among the several factors involved, chronic and neuroinflammation, cellular senescence, genome instability and proteostasis dysregulation are listed [58,59]. Notably, even exogenous factors and toxins may increase the risk of neurodegenerative diseases onset by determining a persistent inflammatory status and oxidative stress [60,61]. Although different in clinical presentation, all these diseases share common features and mechanisms, including abnormal accumulation and aggregation of disease-specific proteins that lead to progressive loss of specific neurons and synapses in distinct brain regions [61,62]. From a clinical point of view, neurodegenerative diseases are characterized by a progressive impairment of the cognitive and/or motor function, the former being the typical pre-clinical condition of AD and frontotemporal dementia [63]. Regarding AD, which represents the main cause of dementia worldwide (60−80%) [64], early symptoms are represented by a progressive memory loss, a decline in cognitive functions, and behavioral signs, including mood changes and depression [65]. The prodromal form of AD is defined as mild cognitive impairment (MCI) and is characterized by alterations of specific cognitive domains among memory, executive function, attention, visuospatial skills and language [66,67]. MCI subjects display a 50% risk in five years for progression of neurocognitive disorders [68]. The current treatments to halt cognitive decline are limited to counteract symptoms and have a positive impact on cognition and behavior only in a transient manner, without affecting the underlying pathology. This current scenario makes the development of alternative strategies to prevent or slow the neurodegeneration an urgent need. Among these approaches, the role of diet as a preventive tool for cognitive decline has been an object of intensive research in the last years.

Many observational and intervention studies suggest a relationship between the administration of polyphenol-rich products, such as berries, grape, cocoa and green tea, and an improvement in cognitive performance [69,70,71,72]. However, not all studies have univocally brought positive results, leaving the open question on the potential applications of polyphenols for cognitive health. First, a recent meta-analysis reported no significant benefit of the Mediterranean diet, whose supplying of polyphenol is about 1g/day [49], on incident cognitive impairment [73]. In addition, an overview of the effect of soy isoflavones concluded that their effects on cognition may be limited and influenced by several factors related to individual features, type of treatment, and the plant itself [74]. Similarly, a meta-analysis of 21 clinical trials indicated that the treatment of MCI patients with Ginkgo biloba extract brought inconsistent results on the cognitive endpoint [75].

Possible explanations for these discrepancies are the previously mentioned bioavailability issues and the inter-individual variability of gut microbiota composition and function, so that the same polyphenols may translate in different effects on people, depending on individual characteristics [76]. In summary, the lack of convincing results from the clinical trials and the above cited criticism make it difficult to draw a solid conclusion on their preventive effects on cognitive status and neurodegeneration.

In this work, we performed a meta-analysis of human trials, in order to help clarify the efficacy of polyphenols with respect to several aspects of cognitive decline. In detail, we focused on healthy elderly or MCI subjects, and we considered the impact of polyphenols on global cognitive health, as well as on specific cognitive domains, including memory, and verbal and executive functions.

### 3.2. Mechanisms of Neuroprotection

Multiple mechanisms are likely to account for polyphenol-induced prevention of age-related neurodegeneration [77,78]. Seminal observations attributed these benefits to the well described radical scavenging activity, allowing the reduction of brain cell damage. Even in the case of neuroprotection, we have to acknowledge that in vitro tests do not always reflect effective antioxidant capacity in humans, as stated above [24]. An additional antioxidant mechanism is the metal-chelating activity, which may prevent the enzymatic production of reactive oxygen species, catalyzed by the metals themselves [22]. This effect is likely to be responsible, at least in part, for the neuroprotective effective of some flavonoids [79]. Recent evidence suggests that polyphenols, in particular flavonoids, may affect specific processes involved in the onset of neurodegenerative disease. Among these, the reduction of neuropathological proteins accumulation [80] and the improvement of synaptic plasticity [81] have been described in animal models of neurocognitive dysfunction treated with physiological doses of polyphenols. Strikingly, the role of polyphenols in improving brain vascular function, thus maintaining cerebral blood flow underpinning cognitive activity, has been well established in young and old adults [82,83].

Less consistent results are obtained in the research for polyphenol-mediated protection of neuroinflammation. The potential anti-inflammatory mechanisms have been described in glial and neuronal cells exposed to supraphysiological concentrations of flavonoids and some studies on humans revealed flavonoid ability to reduce plasma levels of several inflammatory markers [84,85]. However, the anti-inflammatory potential of polyphenols remains uncertain, since these effects were not confirmed in other small trials [86,87]. An additional mechanism explaining the neuroprotective action of polyphenols against AD is the inhibition of cholinesterases, resulting in increased cholinergic activity and improved cognitive performance [88]. This activity has been described for resveratrol, which was able to reduce acetylcholinesterase in specific brain regions of diabetic rats, with a consequent amelioration of memory impairment [89].

The previously described inter-relationships between polyphenols and microbiota also have relevant implication in the setting of neuroprotection. Indeed, intestinal-derived metabolites of polyphenols have been shown to prevent amyloid oligomerization with potential protection against neurodegenerative disorders in rats [90].

### 3.3. Meta-Analysis

#### 3.3.1. Methods

The literature search was performed evaluating both MEDLINE and Embase databases, considering all manuscript published in English-language until November 10, 2018. The following search strategy was performed: ((polyphenol) OR flavonoids) OR polyphenolic compounds) OR Isoflavone) OR flavanol) OR phytoestrogen) OR resveratrol) AND neurocognitive function) OR cognitive function) OR cognition. The literature search was repeated adding the words “administration” and “therapy” using Mesh terms. Inclusion criteria were the same described above. During data extraction, the following endpoints were considered: Wisconsin Card Classification Test (WCST) [91], Reys Auditory Verbal Learning Task (RAVLT) [92], Trail Making test (TMT A and B) [93], Alzheimer’s Disease Assessment Scale-cognitive subscale (ADAS-Cog) [94], Mini-Mental State Examination (MMSE) [95] and Wechsler Adult Intelligence Scale (WAIS) [96]. The meta-analysis of the data extracted from the included studies was conducted as described in the Section 2.3.1.

#### 3.3.2. Results

**Figure 8:**
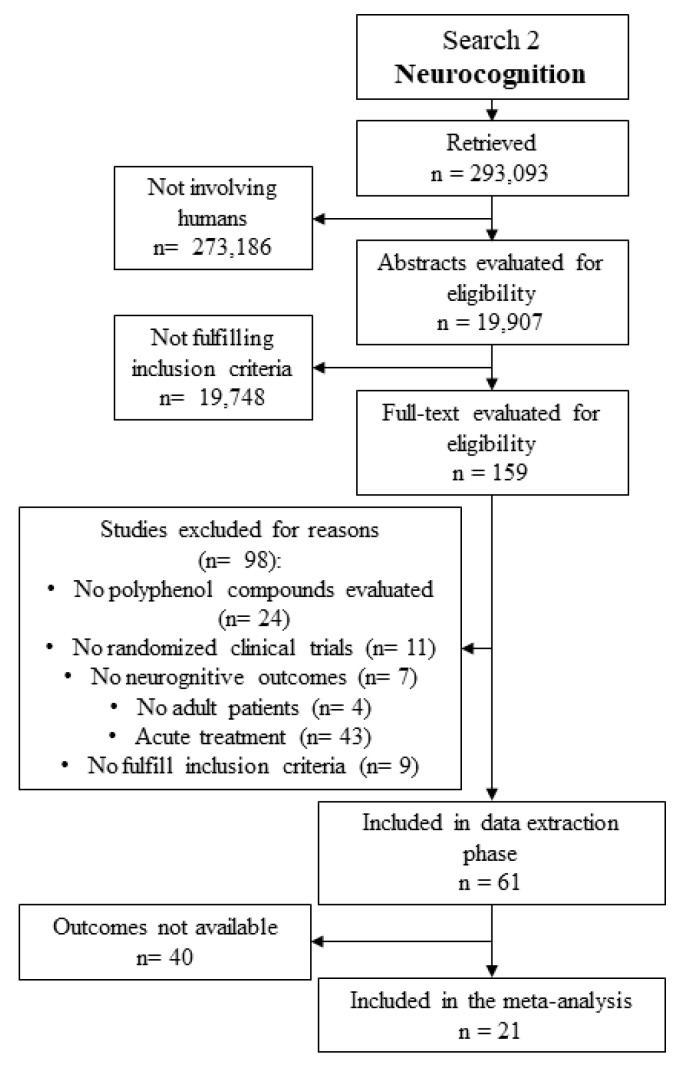
Literature search flow chart for meta-analysis on neurocognitive outcomes.

The literature search detected 293,093 documents. Studies evaluating animal or cellular models were excluded and 19,907 remained for the abstract evaluation. Among these, 61 studies fulfilled inclusion criteria and were further evaluated for data extraction. Neurocognitive endpoints considered for the analysis were available in 21 studies, which were entered in the meta-analysis (Figure 8 and Table 2).

The excluded studies and reason for exclusion are reported in supplementary material (Table S2).

**Figure 9:**
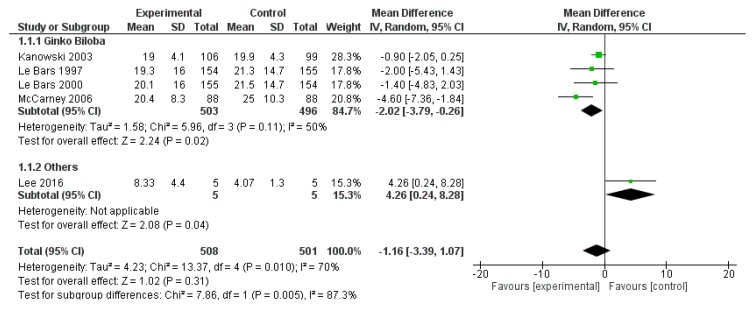
Pooled analysis of the impact of polyphenols on Alzheimer’s Disease Assessment Scale-cognitive subscale (ADAS-Cog).

The ADAS-Cog score was reported in five studies, for a total of 508 patients treated and 501 controls. The overall effects did not show a significant impact of polyphenols on ADAS-Cog (*p* = 0.310). However, the analysis showed a significant effect of Ginkgo biloba on ADAS-Cog, compared to the placebo (mean difference −2.02, 95% CI −3.79; −0.26, *p* = 0.020). (Figure 9).

MMSE was reported in eight studies, for a total of 1868 patients treated and 1856 controls. The overall effect did not show any significant effect of polyphenols on MMSE outcome (*p* = 0.080) (Figure S1).

**Figure 10:**
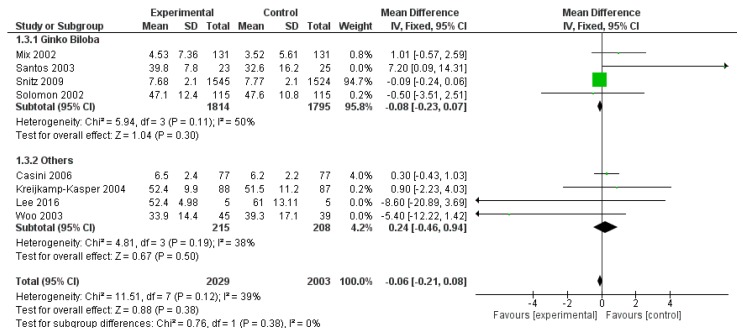
Pooled analysis of the impact of polyphenols on Wechsler Adult Intelligence Scale (WAIS) Digital symbol field.

Considering WAIS, three outcomes were reported in more than three studies: Digital symbol, Block design and Digit Span. Thus, only these three WAIS field were meta-analysed. WAIS digital symbol field was reported in eight studies, for a total of 4032 patients (2029 patients and 2003 controls), without a significant effect of polyphenols (*p* = 0.380) (Figure 10).

Block design field, on the contrary, was evaluated in four studies, for a total of 3389 patients, suggesting a significant reduction (*p* = 0.005), in particular when Ginkgo biloba was used (mean difference −0.43, 95%CI: −0.73; −0.13, *p* = 0.005) (Figure S2).

**Figure 11:**
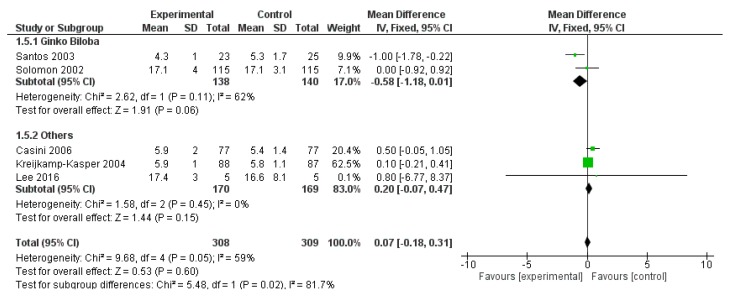
Pooled analysis of the impact of polyphenols on Wechsler Adult Intelligence Scale (WAIS) Digit Span field.

WAIS Digit Span field was reported in five studies for a total of 308 patients and 309 controls. The overall effect was not significantly affected by polyphenol administration (*p* = 0.600) (Figure 11).

**Figure 12:**
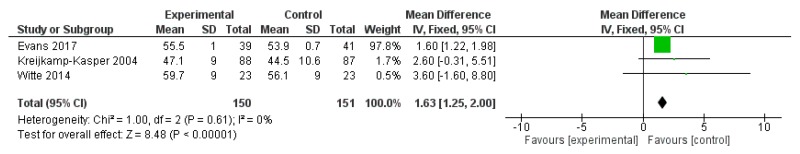
Pooled analysis of the impact of polyphenols on Reys Auditory Verbal Learning Task (RAVLT)**.**

RAVLT was reported in three studies, for a total of 301 subjects (150 treated and 151 controls). Polyphenol administration significantly increased RAVLT immediate score (mean difference 1.63, 95% CI: 1.25; 2.00, *p* < 0.001) (Figure 12).

WCST was reported in four studies for a total of 456 patients. The overall effect was not significantly influenced by polyphenol administration (*p* = 0.070) (Figure S3). Trail making test A was used in seven studies, for a total of 3473 patients. The polyphenol administration did not significantly improve this score (p = 0.620) (Figure S4).

**Figure 13:**
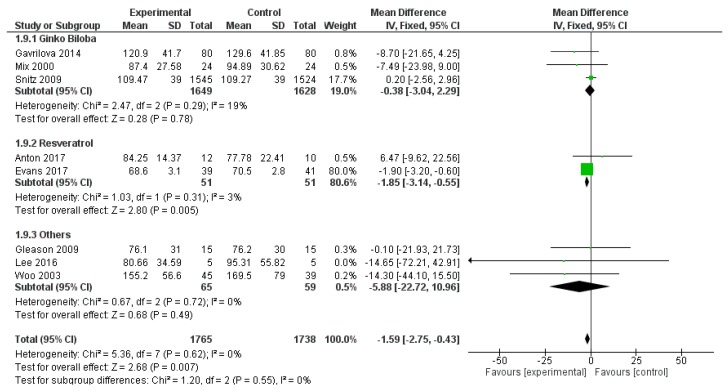
Pooled analysis of the impact of polyphenols on Trail Making Test B.

Similarly, trail making test B was used in eight studies, for a total number of 3503 subjects (1765 patients and 1738 controls). The overall polyphenol effect was statistically significant (mean difference −1.59, 95% CI: −2.75; −0.43, *p* = 0.007), probably for the contribution of resveratrol, evaluated in two single studies (mean difference −1.85, 95% CI: −3.14, −0.55, *p* = 0.005) (Figure 13).

#### 3.3.3. Discussion

In the current meta-analysis, we investigated polyphenol effects on different features of cognitive functions, in order to provide an overall picture of the reported activities. As already specified, our meta-analysis focused on intervention studies using polyphenol-containing food supplements. In particular, the selected studies considered amounts of polyphenols-rich extract up to 480 mg, 300 mg and 100 mg for Gingko biloba, resveratrol and soy isoflavones, respectively. These dosages provide an amount of polyphenols that is very likely to be higher, compared to that reachable by diet [49].

The global cognitive functions, evaluated through MMSE and ADAS-Cog scores, were not significantly improved by polyphenols. The only exception was Gingko biloba, whose intake translated in an improvement on the ADAs-Cog score, one of the most frequently used tests measuring the extent of cognitive decline through the evaluation of language and memory. Our results strengthen the observation of a previous meta-analysis of two studies in which a not statistically significant trend towards improvement in ADAS-Cog score was observed in the Ginkgo biloba group [97]. Similarly, among the different fields analyzed within the battery of the WAIS test, only the block design, often thought as a prototype test for visuospatial ability, was improved by treatment with polyphenols. As for ADAS-Cog, the most significant results were obtained with Gingko biloba extract. It has to be noted that such positive result is driven by the results of one over the three studies analyzed [98]. Thus, this observation requires confirmation with further studies. Nowadays, a possible explanation for the efficacy of Gingko may be attributable to the presence of several active compounds in the extract, such as diterpenes, ginkgolides and a number of flavonoids [99]. All these molecules may act on multiple sites in the central nervous system, all together contributing to attenuating cognitive decline.

By specifically analyzing the impact of polyphenols on distinct cognitive domains, we found a significant improvement in the RAVLT score, which evaluates verbal learning and memory, although the total number of subjects was limited. In particular, the three studies included in the meta-analysis focused on the effect of soy isoflavones and resveratrol and all led to positive results. The observation is consistent with two previous meta-analyses that were specifically aimed at investigating the effect of resveratrol and soy [100,101]. However, the latter study utilized a unique score that took into account the results of all neuropsychological tests together, while in our study we discriminated the effects on specific cognitive domains. On the other hand, our results on resveratrol are inconsistent with those of another recent meta-analysis, in which a beneficial impact has been demonstrated on the mood profile, rather than on memory and cognition performance [102]. A possible explanation for these discrepancies is the selection criteria of the studies (in our case only aged or MCI patients were included) and very high inter-individual differences on resveratrol bioavailability that may result from poor water solubility, chemical instability and difference in hepatic and intestinal metabolism [103].

Another score that was ameliorated by polyphenols was TMT-B, which evaluates executive functions, while no effect was observed on attention, measured by TMT-A. Notably, the analysis was conducted on more than 3000 subjects, making this result clinically relevant. In addition, in this case, the positive effect on the outcome is driven by resveratrol. The superior efficacy of resveratrol in our meta-analysis is in agreement with the beneficial effect on cerebrovascular functions and cognition, demonstrated in a recent trial on post-menopausal women [104].

The main limitation of this meta-analysis is related to the wide number of scores that have been used across the trials to evaluate the impact of polyphenols on cognitive performances. In the effort of analyzing the effect on individual scores, reflecting a particular feature of the cognitive domain, we restricted the analysis to a small number of studies compared to those available in literature. To overcome this criticism, as for cardiovascular markers, there is the necessity to identify the most consolidated markers of neurocognitive improvement, also in order to accomplish to the requests of the European Food Safety Authority (EFSA) for health claims on foods. In this regard, a precise indication of which psychological test is more appropriate for evaluation of central nervous system functions is lacking [105]. However, an ongoing project aims to answer this question in the neurological context [55].

On the other hand, the strength of our work is the strict selection of subjects, which excluded patients already affected by dementia, in order to specifically highlight some potential positive effects of polyphenols in the prevention of cognitive decline. In conclusion, the results of this meta-analysis suggest that polyphenol supplementation might improve specific measures of cognitive status. In addition, among all compounds, stilbene resveratrol appears to be the most active. In the case of Gingko biloba, the reported benefits could be attributed to the whole phytocomplex, where polyphenolic and non-polyphenolic compounds are likely to play an active role.

Given the differences among the studies in terms of patient features and the low number of analyzed studies, as well as the literature inconsistency across several trials, further research is needed to verify whether long-term intake of either single or multiple polyphenols may be a promising strategy for the prevention of cognitive decline in age-related diseases.

## 4. Discussion

The global, objective evaluation of polyphenol health effects presents several points of criticism. One is the existence of almost 10,000 polyphenols with peculiar structures and physiological roles, making the elucidation of their short- and long-term health effects challenging. Second, the recently well-described role of microbiota in the metabolism of polyphenols accounts for different metabotypes, which can differently affect physiological functions, producing high inter-individual differences in the biological response. Third, it is hard to compare data coming from observational/epidemiological studies with intervention trials. While in the former, the global effect is the result of the synergistic interaction of multiple components of the diet, the latter, although limiting the burden of potential confounding factors, may suffer from low adherence to real life conditions, where polyphenols are consumed in small amounts throughout life.

As suggested by Mena in his recent commentary, research on polyphenols requires an urgent, rigorous experimental approach in order to unravel their preventive potential on the development of diseases [10]. According to this, the evaluation of pattern of consumption, metabolites in circulation, inter-individual variations and new biological targets, needs to become pivotal aspects to be considered in every study. As soon as these guidelines are accepted by all reasearchers, data collected in the field of polyphenols and human health will be more realistic.

In the present review, we critically evaluated and meta-analyzed only intervention studies using polyphenol-containing food supplements, finding promising, but not definitive indications of the preventive effects of some polyphenols on cardiovascular disease and cognitive decline.

We are aware of the limitations of this study: the selection of trials with food supplements prevents considerations on the health benefits derived from a naturally polyphenol-enriched diet, where the synergistic effects of different components may appear. On the same trail, the consumption of individual polyphenolic compounds may not produce the same benefits observed in epidemiological studies. In addition, data coming from studies on food supplements, where polyphenol intake is higher than normal, could not be realistic in terms of interaction with drugs or other dietary components. When polyphenols are consumed in drug formulations, they should be considered as nutraceuticals, whose safety should also be addressed, especially if their bioavailability and biological activity are increased.

## 5. Conclusions

Although most polyphenol-based supplements in the market claim beneficial effects based only on in vitro data, the amount and quality of rigorous randomized clinical trials has been increasing for years. However, the long-term effects are yet to be elucidated and studies with hard clinical end points are still lacking. Studies aiming to fully characterize polyphenols pharmacokinetics and their safety are necessary to unravel their potential preventive role in real life.

## Figures and Tables

**Table 1:** Study characteristics after search for cardiovascular effects.

					Study Group		Control Group		
Author	Year	Compound or Extract	Duration	Inclusion Criteria	*n*	Age ± SD	*n*	Age ± SD	Cardiovascular Endpoint
Arazi H	2014	Green tea extract	3 weeks	Hypertensive	8	46.12 ± 5.4	8	48.0 ± 7.1	Blood pressure
Argani H	2016	Red grape seed extract	8 weeks	moderate hyperlipidaemia	35	47.3 ± 9.3	35	46.6 ± 8.4	HDL-C, LDL-C, apolipoprotein A-I, paraoxonase
Atteritano M	2007	Genistein	104 weeks	Osteopenia	198	54.7 ± 0.25	191	54.2 ± 0.19	Lipid status, ICAM-1, VCAM-1
Aubertin-Leheudre M	2008	Isoflavone	26 weeks	Postmenopausal, obese	21	57.1 ± 5.6	18	57.7 ± 5.2	Lipid status
Botden IP	2012	Red wine polyphenols	4 weeks	Healthy subjects	38	61 ± 8	20	61 ± 8	Blood pressure, aortic augmentation index, pulse wave reflection index
Brüll V	2015	Quercetin	6 weeks	Overweight	68	47.4 ± 10.5	68	47.4 ± 10.5	Blood pressure, ET-1, ICAM-1, VCAM-1, hs-CRP, HDL-C, LDL-C, triglycerides
Chan YH	2008	Isoflavone	12 weeks	Ischaemic stroke	50	66.8 ± 9.6	52	65.8 ± 10.3	FMD, hs-CRP, blood pressure, superoxide dismutase
Chiva-Blanch G	2013	Red wine polyphenols	4 weeks	Healthy subjects	67	60 ± 8	67	60 ± 8	HDL-C, LDL-C, apolipoprotein A-I
Choi EY	2015	Onion peel extract	12 weeks	Overweight	34	43.6 ± 9.1	28	42.5 ± 8.9	FMD, Endothelial progenitor cells
Colacurci N	2005	Isoflavone	26 weeks	Postmenopausal	29	55.4 ± 3.7	28	54.9 ± 4	FMD, ICAM-1, VCAM-1, E-selectin
Dower JI	2015	Epicatechin	4 weeks	Healthy subjects	37	66.4 ± 7.9	37	66.4 ± 7.9	Blood pressure
Egert S	2009	Quercetin	6 weeks	Overweight	93	45.1 ± 10.53	93	45.1 ± 10.53	HDL-C, LDL-C, triglycerides, TNF-α
Gliemann L	2013	Resveratrol	8 weeks	cardiovascular risk increased	14	65 ± 1	13	65 ± 1	HDL-C, LDL-C, triglycerides, ICAM-1, VCAM-1
Hale G	2002	Isoflavone	2 weeks	Postmenopausal	16	56.5 ± 4.5	13	58 ± 7.2	FMD
Hallund J	2006	Isoflavone	8 weeks	Postmenopausal	28	57 ± 5	28	57 ± 5	FMD, ET-1, blood pressure,
Hanson LN	2006	Isoflavone	6 weeks	Postmenopausal	14	60 (60–70)	14	56 (49–70)	hs-CRP
Hassellund SS	2013	Anthocyanins	4 weeks	Hypertension	27	41 ± 3	27	41 ± 3	HDL-C, LDL-C, triglycerides
Howes JB	2000	Red-clover isoflavones	4 weeks	Type 2 diabetes mellitus	75	58 ± 7.3	75	58 ± 7.3	HDL-C, LDL-C, triglycerides
Howes JB	2003	Red-clover isoflavones	4 weeks	Type 2 diabetes mellitus	19	62 ± 2	19	62 ± 2	Blood pressure
Jayagopal V	2002	Isoflavone	12 weeks	Postmenopausal, type 2 diabetes mellitus	32	62.5 ± 6.8	32	62.5 ± 6.8	HDL-C, LDL-C, triglycerides
Lockyer S	2017	Phenolic-rich olive leaf extract	12 weeks	Healthy subjects	30	45.3 ± 12.7	30	45.3 ± 12.7	HDL-C, LDL-C, triglycerides, blood pressure
Mainini G	2013	Isoflavone	12 weeks	Postmenopausal	67	54.6 ± 5	61	54.6 ± 5	HDL-C, LDL-C, triglycerides, apolipoprotein A-I
Nestel P	2007	Trans-tetrahydrodaidzein	5 weeks	Postmenopausal, overweight	25	57 ± 7	25	57 ± 7	Blood pressure, HDL-C, LDL-C, triglycerides
Nogueira LP	2017	Green tea extract	4 weeks	Hypertension, overweight	20	41.1 ± 8.4	20	41.1 ± 8.4	Blood pressure, HDL-C, LDL-C, triglycerides
Panahi Y	2015	Curcuminoid-piperine combination	8 weeks	Metabolic syndrome	59	44.8 ± 8.7	58	43.5 ± 9.7	hs-CRP
Pipe EA	2009	Soy protein isolate	4 weeks	Type 2 diabetes mellitus	29	60.1 ± 9.6	29	60.1 ± 9.6	HDL-C, LDL-C, triglycerides, apolipoprotein A-I
Saarenhovi M	2017	Apple polyphenol extract	4 weeks	Hypertension	13	55.6 ± 7.9	13	54.9 ± 6.4	FMD
Teede HJ	2003	Biochanin	6 weeks	Healthy subjects	80	54 ± 0.7	80	54 ± 0.7	Blood pressure, FMD, arterial stiffness
Tomé-Carneiro J	2013	Resveratrol	26 weeks	cardiovascular risk increased	25	60 ± 12	25	58 ± 9	Apolipoprotein A-I, HDL-C, LDL-C, triglycerides
Trautwein EA	2010	Black tea flavonoids	12 weeks	Healthy subjects	31	50.1 ± 3.6	35	46.8 ± 7.1	HDL-C, LDL-C, triglycerides
Valls RM	2016	Oligopin	5 weeks	Hypertension	21	NA	21	NA	HDL-C, LDL-C, triglycerides, blood pressure
Verhoeven MO	2007	Isoflavone	12 weeks	Postmenopausal	56	54.1 ± 4.6	59	53.8 ± 4.4	HDL-C, LDL-C, triglycerides, lipoprotein, hs-CRP
Weseler AR	2011	Flavanols	8 weeks	Smokers	15	46 (30–58)	13	(30–60)	HDL-C, LDL-C, triglycerides, hs-CRP
Xie L	2017	Berry polyphenol	12 weeks	Smokers	25	32.6 ± 2.6	24	37.4 ± 3.0	HDL-C, LDL-C, triglycerides

ET-1: endothelin-1; FMD: flow mediated dilation; HDL-C: high-density lipoprotein-cholesterol; hs-CRP: high sensitivity C-reactive protein; ICAM-1: intercellular adhesion molecule-1; LDL-C: low-density lipoprotein-cholesterol; VCAM-1: vascular cell adhesion molecule-1; TNF-α: tumor necrosis factor-α; NA: not available; SD: standard deviation.

**Table 2:** Study characteristics after search for neurocognitive effects.

					Study Group		Control Group		
Author	Year	Compound or Extract	Duration	Inclusion Criteria	*n*	Age ± SD	*n*	Age ± SD	Neurocognitive Endpoints
Dai CX	2018	Ginkgo biloba extract	12 weeks	Depression	68	66..48 ± 4.12	68	66.82 ± 3.35	HAMD, WCST
Anton SD	2017	Resveratrol	12 weeks	Healthy subjects	12	73.17 ± 2.08	10	73.3 ± 2.06	Trail Making, Digits Forward and Backward, Erikson-Flanker, Controlled Oral Word Association, Hopkins Verbal Learning Test-Revised, and Task Switching
Evans HM	2017	Resveratrol	14 weeks	post-menopausal women	39	61.5 ± 1.1	40	61.5 ± 1.2	RAVLT, trail making test, visual spatial working memory, semantic memory tests, Profile of Mood States
Lee J	2016	Grape formulation	24 weeks	Cognitive impairment	5	71.2 ± 2.06	5	73.2 ± 5.02	ADAS-Cog, Hopkins VLT, FAS, trail making test, WCST-64, WAIS-IIIWATR, MMSE, HDRS, HARS, CIBIC-Plus
Gleason CE	2015	Soy isoflavones	25 weeks	Healthy subjects	32	75.7 ± 7.7	33	76.8 ± 6.8	MMSE, verbal memory, Mazes, trail making test, stroop color word test, fluency, visual memory, visual motor, GDS, POMS
Witte AV	2014	Resveratrol	26 weeks	Healthy subjects	23	64.8 ± 6.8	23	63.7 ± 5.3	Auditory Verbal Learning Test, MRI
Gavrilova SI	2014	Ginkgo biloba extract	24 weeks	Cognitive impairment	80	65 ± 7	79	63 ± 7	CAMCOG, MMSE, Neuropsychiatric Inventory, State-Trait Anxiety Inventory, Geriatric Depression Scale, Trail-Making Test
Henderson VW	2012	Isoflavone-rich soy protein	120 weeks	post-menopausal women	175	61 ± 7	175	61 ± 7	Neuropsychological battery, Wechsler Test of Adult Reading, Center for Epidemiological Studies Depression scale (CES-D)
Kaschel R	2011	Ginkgo biloba extract	6 weeks	Healthy subjects	88	45–56	89	45–56	Memory tests
Snitz BE	2009	Ginkgo biloba extract	312 weeks	Healthy subjects	1545	79.1 ± 3.3	1524	79.1 ± 3.3	MMSE, ADASCog
Gleason CE	2009	Isoflavone-rich soy protein	24 weeks	Healthy subjects	15	73.0 ± 7.9	15	74.3 ± 6.3	Buschke Selective Reminding test, VSL, Boston Naming test, FAS, Grooved Pegboard, Stroop Color Word test, Mazes, Trail Making Test
Stough C	2008	Bacopa monnieri extract	12 weeks	Healthy subjects	33	41.6 ± 13.4	29	44.3 ± 11.3	CDR, RVIP
McCarney R	2008	Ginkgo biloba extract	24 weeks	Dementia	88	79.3 ± 7.77	88	79.7 ± 7.53	ADAS-Cog, QOL-AD
Woelk H	2007	Ginkgo biloba extract	4 weeks	Anxiety disorder	60	47.6 ± 11.7	37	46.7 ± 13	HAMA, CGI-C, EAAS
Casini ML	2006	Isoflavone-rich soy protein	24 weeks	post-menopausal women	77	49 ± 4.3	77	50 ± 3.9	Digit Symbol Test, WAIS, HRSD
Kreijkamp-Kaspers S	2004	Isoflavone-rich soy protein	52 weeks	post-menopausal women	88	66.5 ± 4.7	87	66.7 ± 4.8	MMSE, RAVLT, WAIS
Kanowski S	2003	Ginkgo biloba extract	24 weeks	Degenerative dementia	106	72 ± 10	99	72 ± 10	ADAS-Cog, MMSE, Clinical Global Impression of Change
Santos RF	2003	Ginkgo biloba extract	32 weeks	Healthy subjects	23	60–70	25	60–70	WAIS, Wechsler Memory Scale revised (WMS-R), WCST, Toulouse-Pièron Concentrated Attention
Woo J	2003	Pueraria lobata (isoflavone)	12 weeks	post-menopausal women	45	57.4 ± 4.6	39	57.2 ± 4.8	SF-36, HKLT, MMSE, WAIS; Trail making test
Kritz-Silverstein D	2003	Isoflavone-rich soy protein	24 weeks	post-menopausal women	27	55–74	26	55–74	Trails A and B, category fluency, and logical memory and recall
Mix JA	2002	Ginkgo biloba extract	6 weeks	Healthy subjects	131	66.97 ± 6.12	131	68.6 ± 6.96	SRT, WAIS, WCST
Solomon PR	2002	Ginkgo biloba extract	6 weeks	Healthy subjects	115	68.7 ± 4.7	108	69.9 ± 5.4	WAIS, WMS, CVLT, Boston Naming test
File SE	2001	Isoflavone-rich soy protein	10 weeks	Healthy subjects	37	27.1 ± 3.2	37	23.9 ± 2.2	Stocking Cambridge Test, PASAT, WMS
Mix JA	2000	Ginkgo biloba extract	6 weeks	Healthy subjects	24	67.5 ± 9.23	24	68.65 ± 6.95	MMSE, SCWT, Trail Making Test, WMS
Le Bars PL	2000	Ginkgo biloba extract	26 weeks	Degenerative dementia	154	69 ± 10	155	69 ± 10	ADAS-Cog, Geriatric Evaluation by Relative’s Rating Instrument(GERRI) and Clinical Global Impression of Change
Le Bars PL	1997	Ginkgo biloba extract	52 weeks	Degenerative dementia	166	69 ± 10	161	69 ± 10	ADAS-Cog, Geriatric Evaluation by Relative’s Rating Instrument

ADL-IS: Activities of Daily Living International Scale; AOT: Auditory order threshold test; BLMD: Bond and Lader Mood Scale; CANTAB: Cambridge Neuropsychological Test Automated Battery; DCT-G: Digit connection test–G; DMTS: Delayed Matching To Sample Test; HADS: Hospital Anxiety and Depression Scales; HKLT: Hong Kong List-Learning Test; ITVS: Test’s increment threshold for visual stimuli; MMSE: Mini Mental State Examination; NPI: Neuropsychiatric Inventory; PASAT: Paced Auditory Serial Addition Task; POMS: profile of mood states ; RAVLT: Rey Auditory Verbal Learning Test; SDS: self-rating depression scale including; SIS-M: subjective intensity scale-mood; SMS: Sensorimotor synchronization test; SRT: Selective Reminding Test; SWM: spatial working memory; WMS: Weschler Memory Scale; SD: standard deviation.

## Supplementary Materials

Supplementary materials can be found at http://www.mdpi.com/1422-0067/20/2/351/s1.

## Abbreviations

CVD	Cardiovascular disease
LDL-C	Low density lipoprotein cholesterol
HDL-C	High density lipoprotein cholesterol
FMD	Flow mediated dilation
NO	Nitric oxide
TNF-α	Tumor necrosis factor alpha
hs-CRP	High-sensitive C-reactive protein
HOMA-AD	Homeostasis model assessment-adiponectin
HOMA-IR	Homeostasis model assessment of insulin resistance
AD	Alzheimer’s disease
MCI	Mild cognitive impairment
WCST	Wisconsin card classification test
RAVLT	Reys auditory verbal learning task
TMT	Trail making test
ADAS-Cog	Alzheimer’s Disease assessment scale-cognitive subscale
MMSE	Mini-mental state examination
WAIS	Wechsler adult intelligence scale
