# Supplementary material for: Polyphenol Health Effects on Cardiovascular and Neurodegenerative Disorders: A Review and Meta-Analysis
Source: Int J Mol Sci. 2019 Jan 16;20(2):351. doi: 10.3390/ijms20020351 (PMC6359281; doi:10.3390/ijms20020351)
Supplement: Supplementary file 1 [file ijms-20-00351-s001.pdf]

Table 1. Studies excluded from the meta-analysis on cardiovascular effects.

						Study group		Control group				
**Author**	**Year**	**Compound**	**Duration**	**Patients' gender**	**Inclusion criteria**	**n**	**Age (± SD)**	**n**	**Age (± SD)**	**Primary endpoint**	**Cardiovascular endpoint**	**Reason for exclusion**
Brüll V	2017	Quercetin	6 weeks	Both	Metabolic syndrome	68	47.4 ± 10.5	68	47.4 ± 10.5	Anti-inflammatory effects	Blood pressure, ET-1, ICAM-1, VCAM-1, hs-CRP, HDL-C, LDL-C, triglycerides	Duplicated
Carmignani LO	2014	Isoflavone	16 weeks	Female	Postmenopausal	20	52.9 ± 3.4	20	50.9 ± 3.4	Cardiovascular health	Blood pressure, HDL-C-C, LDL-C-C, triglycerides	Data not reported
Chedraui P	2008	Trifolium pratense-derived isoflavone	12 weeks	Female	Overweight	31	51.3 ± 3.3	22	51.2 ± 3.9	Lipid status	HDL-C, LDL-C, triglycerides	Data not reported
Chuengsamarn S	2014	Curcumin	26 weeks	Both	Type 2 diabetes mellitus	107	59.2 ± 1.1	106	59.6 ± 1.0	Atherosclerosis	Triglycerides, pulse wave velocity, adiponectin	Data not reported
Dent SB	2001	Isoflavone-supplemented probiotic soy product	24 weeks	Female	Postmenopausal	69	50.2 ± 3.6	69	50.2 ± 3.6	Lipid status	HDL-C, LDL-C, triglycerides	Data not reported
Dower JI	2015	Epicatechin	4 weeks	Both	Healthy subjects	37	66.4 ± 7.9	37	66.4 ± 7.9	Endothelial function	E-selectin	Data not reported
Fenercioglu AK	2010	Polyphenol-containing antioxidants	12 weeks	Both	Type 2 diabetes mellitus	56	53.51 ± 6.8	58	53.9 ± 1.2	Lipid peroxidation	HDL-C, LDL-C, triglycerides	Data not reported
Rahbar AR	2015	Red grape polyphenol	8 weeks	Both	Hypercholesterolemia	46	50.5 ± 9.7	23	52.5 ± 11.5	Lipid status	HDL-C, LDL-C, triglycerides	Data not reported
Tomé-Carneiro J	2012	Resveratrol	26 weeks	Both	Cardiovascular risk increased	25	60 ± 12	25	58 ± 9	Lipid status	Apolipoprotein A-I, HDL-C, LDL-C, triglycerides	Duplicated

ET-1: endothelin-1; FMD: flow mediated dilation; HDL-C: high-density lipoprotein-cholesterol; hs-CRP: high sensitivity C-reactive protein; ICAM-1: Intercellular adhesion molecule-1; LDL-C: low-density lipoprotein-cholesterol; SD: standard deviation; VCAM-1: vascular cell adhesion molecule-1.

Table 2. studies excluded from the meta-analysis on neuro-cognitive effects.

Author	Year	Polyphenol compound	Duration	Patients' gender	Inclusion criteria	Study group n	Study group Age (± SD)	Control group n	Control group Age (± SD)	Aim of the study	Neurocognitive endpoints	Reason for exclusion
Whyte AR	2018	Wild blueberry Powder	24 weeks	Both	Self-reported memory complaints	30	65–80	30	65–80	Cognitive function	RAVLT, visual episodic memory was assessed using an object recognition task, Corsi Blocks task	RAVLT not reported as number
Miller MG	2017	Blueberry extract	13 weeks	Both	Healthy subjects	20	60–75	20	60–75	Cognitive function	GDS, POMS, TST, trail-making test, California Verbal Learning Test, digit span task, virtual version of the Morris Water Maze, Attention Network Task	No endpoints reported
Boespflug EL	2018	Blueberry extract	16 weeks	Both	Cognitive impairment	8	80.4 ± 7.3	8	75.5 ± 4.8	Cognitive function	BOLD, MRI, working memory task	No endpoints reported
Herrlinger KA	2018	Spearmint extract	12 weeks	Both	Cognitive impairment	30	59.1 ± 1.0	30	58.2 ± 1.2	Cognition, sleep and mood	CDR, LSEQ, POMS	No endpoints reported
Bowtell JL	2017	Blueberry extract	12 weeks	Both	Healthy subjects	12	67.5 ± 3.0	14	69 ± 3.3	Cognitive function	MRI, Groton maze learning task, international shopping list task, back test	No endpoints reported
Gschwind YJ	2017	Ginkgo biloba extract	52 weeks	Both	Cognitive impairment	25	67.8 ± 8.3	25	69 ± 8.6	Gait stability	Spatio-temporal gait parameters	No endpoints reported
Evans HM	2016	Resveratrol	14 weeks	Female	post-menopausal women	NA	NA	NA	NA	Cognitive function	Rey Auditory Verbal Learning Test, Cambridge Semantic Memory Battery, the Double Span and the Trail Making Task.	Methodological trial
Beck SM	2016	Ginkgo biloba extract	8 weeks	Both	Self-reported memory complaints	31	57.5 ± 4.6	30	57.1 ± 4.4	Cognitive function	Task-set-switching, response-inhibition, delayed response, prospective-memory, task-related fMRI-BOLD-signals and the Trier Social Stress-Test	No endpoints reported
Wightman EL	2015	Resveratrol	7 weeks	Both	Healthy subjects	30	18–30	30	18–30	Cognitive function	Food consumption questionnaire, GHQ, POMS, PSQI	No endpoints reported
Wang LP	2015	Ginkgo biloba extract and Ginseng	12 weeks	Both	Vascular cognitive impairment	40	60–75	40	60–75	Cognitive function	MoCA, and transcranial Doppler US	No endpoints reported
St John JA	2014	Isoflavone-rich soy protein	132 weeks (96-164)	Female	post-menopausal women	150	61 ± 7	150	61 ± 7	Cognitive function	Wechsler Test of Adult Reading, Center for Epidemiological Studies Depression scale (CES-D)	No endpoints reported

Pase MP	2013	Cocoa polyphenols	4 weeks	Both	Healthy subjects	25	51.15 ± 7.88	22	52.98 ± 7,65	Cognitive function and mood	Working Memory, Episodic Secondary Memory, Power of Attention, Speed of Memory and Continuity of Attention, Bond and Lader Visual Analogue Scales	No endpoints reported
Mohamed S	2013	Catechin-rich oil palm leaf extract	8 weeks	Both	Healthy subjects	15	NA	15	NA	Cognitive function	Short-term memory ability, Processing speed ability, Spatial visualisation learning ability	No endpoints reported
Zhang SJ	2012	Ginkgo biloba extract	12 weeks	Both	Vascular cognitive impairment	40	66.5 ± 5.6	40	66.5 ± 5.6	Cognitive function	Montreal cognitive assessment (MoCA) and transcranial Doppler US	No endpoints reported
Herrschaft H	2012	Ginkgo biloba extract	24 weeks	Both	Patients with mild to moderate dementia	200	65.1 ± 8.8	202	64.9 ± 9.4	Cognitive function	NPI, SKT, ADCS, Verbal Fluency Test, ADL-IS,, DEMQOL	No endpoints reported
Maki PM	2009	Red clover, black cohosh	52 weeks	Female	post-menopausal women	49	51.6 ± 5.1	17	52.6 ± 4.3	Cognitive function	CVLT, LMT, BVRT, PANAS	No endpoints reported
DeKosky ST	2008	Ginkgo biloba extract	312 weeks	Both	Cognitive impairment	1545	79.1 ± 3.3	1524	79.1 ± 3.3	Dementia and Alzheimer disease incidence	CVLT, Rey Ostermieth Figure, WAIS, Trail making test, stroop color	Only results about adverse events
Ryan J	2008	Pycnogenol	12 weeks	Both	Healthy subjects	49	66.9 ± 5.3	52	68.4 ± 5.6	Cognitive function	CDR, WASI	No endpoints reported
Dodge HH	2008	Ginkgo biloba extract	168 weeks	Both	Healthy subjects	60	87.14 ± 2.14	58	87.43 ± 2.22	Risk of dementia progression	MMSE	Only regression analyses reported
Burns NR	2006	Ginkgo biloba extract	12 weeks	Both	Healthy subjects	46	61.2 ± 5.7	47	62.2 ± 5.3	Cognitive function	Woodcock–Johnson Psycho-Educational Battery-Revised, 'odd-man-out' reaction time (OMO) task	No endpoints reported
File SE	2005	Isoflavone-rich soy protein	6 weeks	Female	post-menopausal women	25	57.4 ± 0.7	25	58.6 ± 0.8	Cognitive function	Green climateric scale, HADS, BLMD	No endpoints reported
Elsabagh S	2005	Ginkgo biloba extract	6 weeks	Both	Healthy subjects	20	22.3 ± 0.3	20	21.7 ± 0.4	Cognitive function	HADS, SWM, CANTAB, PASAT	No endpoints reported

Elsabagh S	2005	Ginkgo biloba extract	6 weeks	Female	post-menopausal women	45	55.3 ± 0.6	42	55.5 ± 0.6	Cognitive function	HADS, Green Climateric Scale, PASAT	No endpoints reported
Hartley DE	2004	Ginkgo biloba and Ginseng	12 weeks	Female	post-menopausal women	30	58.4 ± 1.0	27	57.4 ± 0.7	Cognitive function and mood	HADS, PASAT	No endpoints reported
Howes JB	2003	Extract of aglycone isoflavones from red clover	24 weeks	Female	post-menopausal women	19	62 ± 2	19	62 ± 2	Cognitive function	Test of visual-spatial intelligence, verbal memory test, digit recall test	No endpoints reported
Mattes RD	2004	Ginkgo biloba extract	13 weeks	Both	Healthy subjects	21	24.1 ± 1.2	18	23.1 ± 1.3	Alertness and chemosensory function	Profile of Mood States, letter cancellation-task	No endpoints reported
Kok L	2004	Isoflavone-rich soy protein	52 weeks	Female	post-menopausal women	100	66.6 ± 4.8	102	66.8 ± 4.7	Cognitive function	RAVLT, WAIS, MMSE, Geriatric Depression Scale	Only study design description
Duffy R	2003	Isoflavone-rich soy protein	12 weeks	Female	post-menopausal women	18	58.8 ± 1.1	15	56.8 ± 1.0	Cognitive function	HADS, DMTS	No endpoints reported
Cieza A	2003	Ginkgo biloba extract	4 weeks	Both	Healthy subjects	34	56.5 ± 3.8	32	56.3 ± 3.8	Cognitive function	ITVS, DCT-G, POMS, SIS-M, SDS, Finger tapping test-personal tempo, Finger tapping test-speed, Auditory choice reaction time, Color word test, AOT, SMS	No endpoints reported
Stough C	2001	Ginkgo biloba extract	4 weeks	Both	Healthy subjects	25	18-40	25	18-40	Cognitive function	Trail Making Test, RAVLT, WAIS	Only study design description
Wesnes KA	2000	Ginkgo biloba/Panax ginseng	12 weeks	Both	Healthy subjects	125	38–66	125	38–66	Cognitive function	CDR	Only study design description
Wesnes KA	1997	Ginkgo biloba extract	12 weeks	Both	Neurasthenic complaints	48	40–65	16	40–65	Cognitive function	CDR, Computerized assessment system, Vienna Determination Unit	No endpoints reported
Rai GS	1991	Ginkgo biloba extract	24 weeks	Both	Memory impairment	12	73.4 ± 7.25	15	78.3 ± 5.93	Cognitive function	MMSE, Kendrick Battery for the detection of dementia	No endpoints reported

ADL-IS: Activities of Daily Living International Scale; AOT: Auditory order threshold test; BLMD: Bond and Lader Mood Scale; CANTAB: Cambridge Neuropsychological Test Automated Battery; DCT-G: Digit connection test–G; DMTS: Delayed Matching To Sample Test; HADS: Hospital Anxiety and Depression Scales; HKLT: Hong Kong List-Learning Test; ITVS: Test's increment threshold for visual stimuli; MMSE: Mini Mental State Examination; NA: not available; NPI: Neuropsychiatric Inventory; PASAT: Paced Auditory Serial Addition Task; POMS: profile of mood states ; RAVLT: Rey Auditory Verbal Learning Test; SD: standard

deviation; SDS: self-rating depression scale including; SIS-M: subjective intensity scale-mood; SMS: Sensorimotor synchronization test; SRT: Selective Reminding Test ; SWM: spatial working memory; WMS: Weschler Memory Scale.

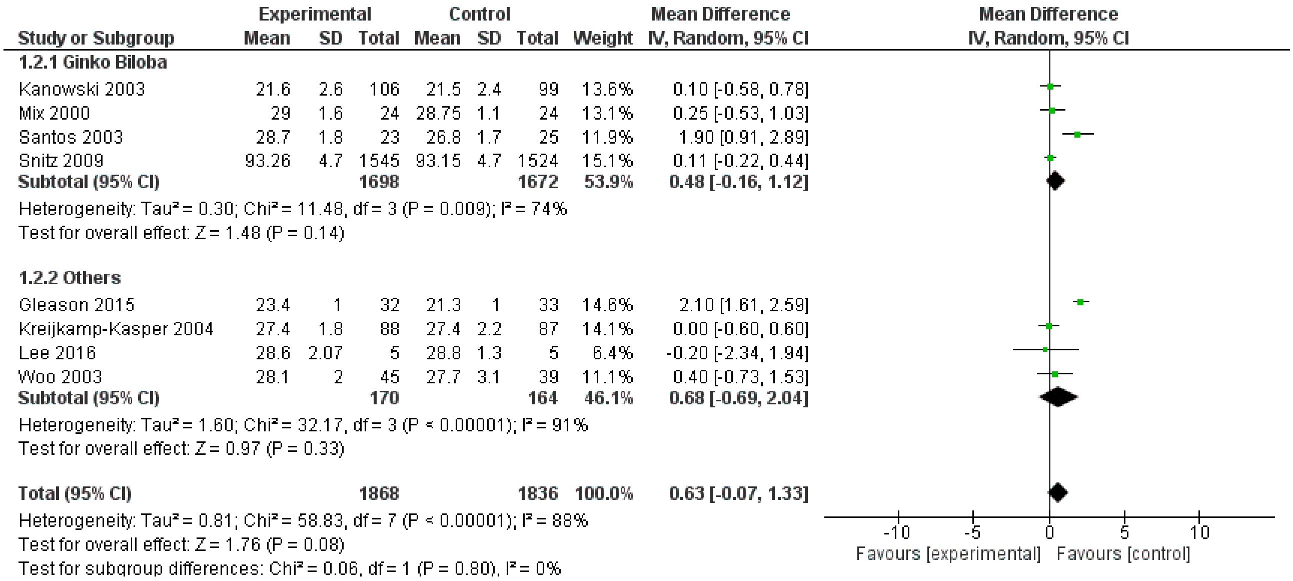

Figure 1. Pooled analysis of the impact of polyphenols on Mini-Mental State Examination (MMSE).

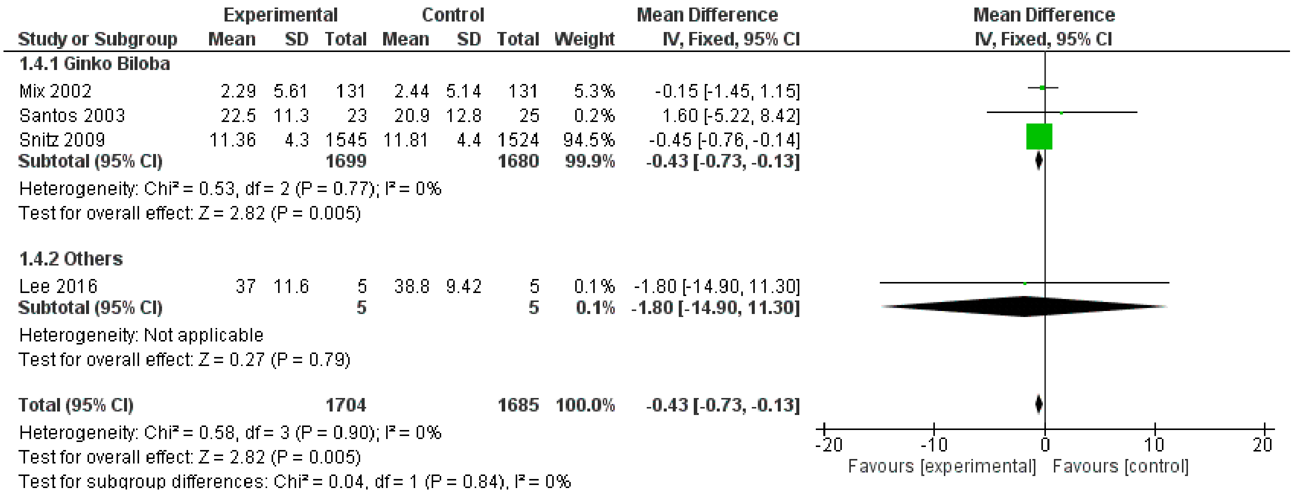

Figure 2. Pooled analysis of the impact of polyphenols on Wechsler Adult Intelligence Scale (WAIS) Block Design field.

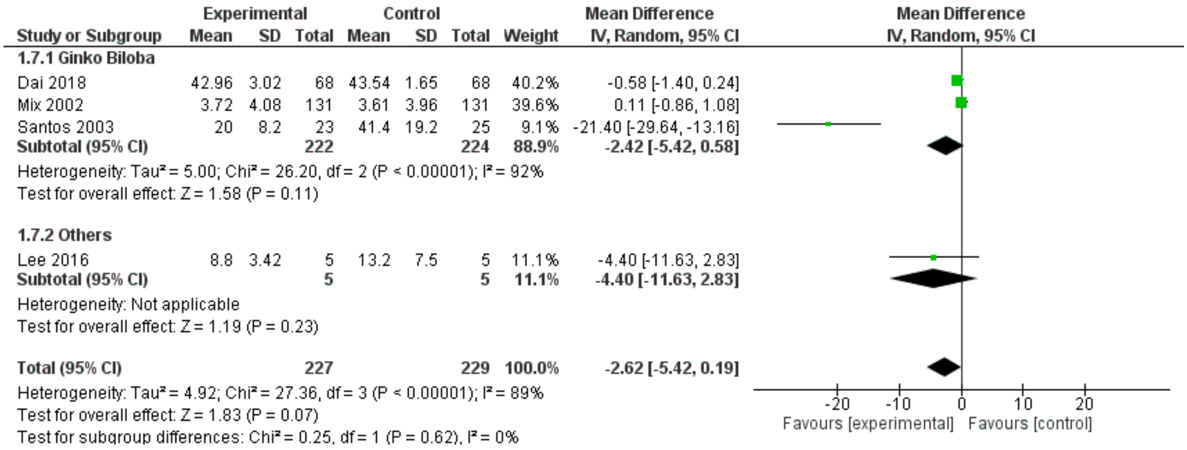

Figure 3. Pooled analysis of the impact of polyphenols on Wisconsin Card Classification Test (WCST).

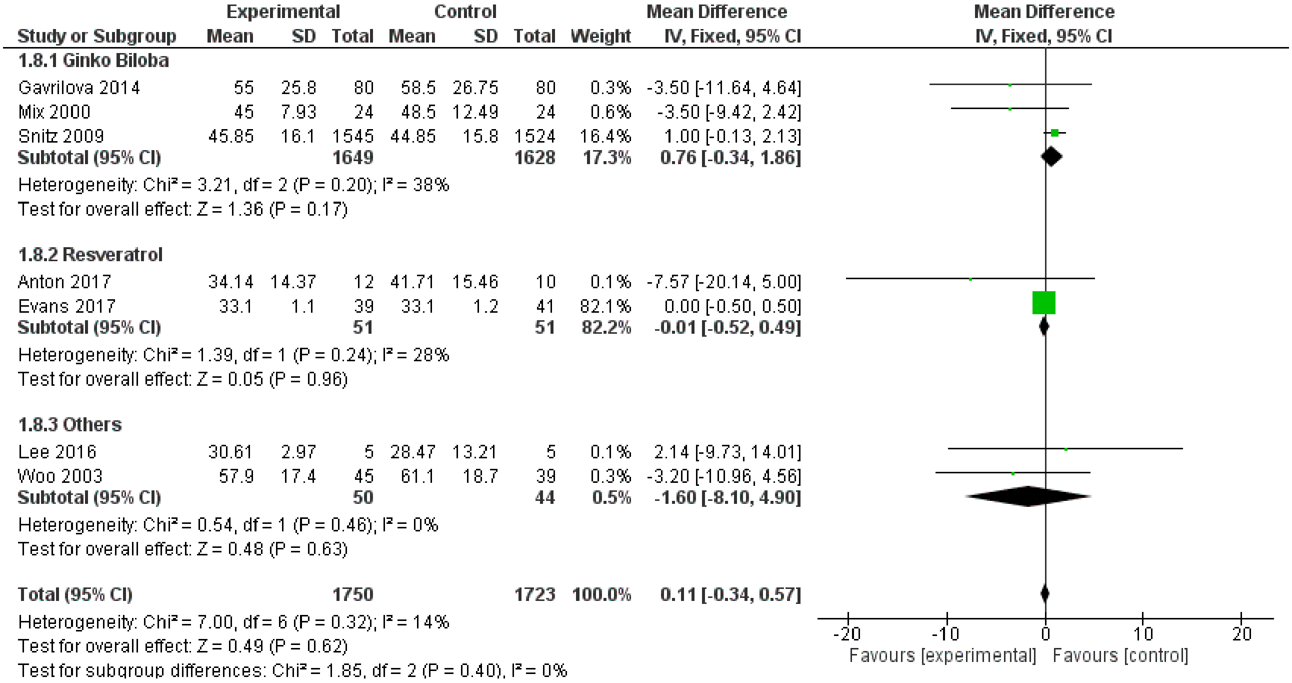

Figure 4. Pooled analysis of the impact of polyphenols on Trail Making Test A.
